# Contributing to Global Health: Development of a Consensus-Based Whole Systems Research Strategy for Anthroposophic Medicine

**DOI:** 10.1155/2019/3706143

**Published:** 2019-11-12

**Authors:** G. S. Kienle, E. Ben-Arye, B. Berger, C. Cuadrado Nahum, T. Falkenberg, G. Kapócs, H. Kiene, D. Martin, U. Wolf, H. Szöke

**Affiliations:** ^1^Institute for Applied Epistemology and Medical Methodology, Witten/Herdecke University, Freiburg, Germany; ^2^Center for Complementary Medicine, Institute for Infection Prevention and Hospital Epidemiology, Medical Center, Faculty of Medicine, University of Freiburg, Freiburg, Germany; ^3^Integrative Oncology Program, Oncology Service, Lin Medical Center, Clalit Health Services, Rappaport Faculty of Medicine, Technion, Haifa, Israel; ^4^Institute for Integrative Medicine, Witten/Herdecke University, Freiburg, Germany; ^5^Program of Health Policy, Systems and Management, School of Public Health, University of Chile, Santiago, Chile; ^6^Department of Neurobiology Care Sciences and Society, Division of Nursing, Research Group Integrative Care, Karolinska Institutet, Stockholm, Sweden; ^7^Department of Psychiatry and Psychiatric Rehabilitation, Saint John Hospital, Budapest, Hungary; ^8^Integrative and Anthroposophic Medicine, Institute for Integrative Medicine, Witten/Herdecke University, Freiburg, Germany; ^9^University of Bern, Institute of Complementary and Integrative Medicine, Bern, Switzerland; ^10^Department of CAM, Faculty of Health Sciences, University of Pecs, Pilisszentkereszt, Hungary

## Abstract

**Background:**

Whole medicine and health systems like traditional and complementary medicine systems (T&CM) are part of healthcare around the world. One key feature of T&CM is its focus on patient-centered and multimodal care and the integration of intercultural perspectives in a wide range of settings. It may contribute to good health and well being for people as part of the Sustainable Development Goals of the United Nations. The authentic, rigorous, and fair evaluation of such a medical system, with its inherent complexity and individualization, imposes methodological challenges. Hence, we propose a broad research strategy to test and characterize its possible contribution to health.

**Methods:**

To develop a research strategy for a specific T&CM system, Anthroposophic Medicine (AM), applying multimodal integrative healthcare based on a four-level concept of man, we used a three-phase consensus process with experts and key stakeholders, consisting of (1) premeeting methodological literature and AM research review and interviews to supplement or revise items of the research strategy and tailor them to AM research, (2) face-to-face consensus meetings further developing and tailoring the strategy, and (3) postmeeting feedback and review, followed by finalization.

**Results:**

Currently, AM covers many fields of medical specialties in varied levels of healthcare settings, such as outpatient and inpatient; primary, secondary, and tertiary care; and health education and pedagogy. It is by definition integrated with conventional medicine in the public healthcare system. It applies specific medicines, nursing techniques, arts therapies, eurythmy therapy, rhythmical massage, counseling, and psychotherapy, and it is provided by medical doctors, nurses, therapists, midwives, and nutritionists. A research strategy authentic to this level of complexity should comprise items with a focus on (I) efficacy and effectiveness, divided into (a) evaluation of the multimodal and multidisciplinary medical system as a whole, or of complex multimodal therapy concept, (b) a reasonable amount of methodologically rigorous, confirmatory randomized controlled trials on exemplary pharmacological and nonpharmacological therapies and indications, (c) a wide range of interventions and patient-centered care strategies with less extensive formats like well-conducted small trails, observational studies, and high-quality case reports and series, or subgroup analyses from whole-system studies, or health service research; (II) safety; (III) economics; (IV) evidence synthesis; (V) methodologic issues; (VI) biomedical, physiological, pharmacological, pharmaceutical, psychological, anthropological, and nosological issues as well as innovation and development; (VI) patient perspective and involvement, public needs, and ethics; (VII) educational matters and professionalism; and (IX) disease prevention, health promotion, and public health.

**Conclusion:**

The research strategy extends to and complements the prevailing hierarchical system by introducing a broad “evidence house” approach to evaluation, something many health technology assessment boards today support. It may provide transparent and comprehensive insight into potential benefits or risks of AM. It can serve as a framework for an evidence-informed approach to AM for a variety of stakeholders and collaborating networks with the aim of improving global health.

## 1. Background

Traditional and complementary medicine (T&CM) is broadly and increasingly used around the world [1]. The wide use is related to cultural aspects and health belief models and to the needs of patients for “whole person care.” T&CM has a strong focus in health maintenance and disease prevention but is also frequently applied for chronic noncommunicable diseases (NCDs). Former WHO general director Margret Chan regarded T&CM as an often-underestimated part of health services, particularly with regard to addressing the challenges of chronic NCDs [1–3] that reach epidemic proportions worldwide, accounting for two-thirds of all deaths. Chronic NCDs have a huge economic impact and lead to high morbidity and disability [4, 5]. Risk factors are mainly lifestyle-related [6] and are associated with the increasing globalization. To address this enormous health challenge, a wider perspective may be sensible: an integration of successful health-supporting strategies and treatments from conventional medicine and T&CM, embedded in transcultural understanding and collaboration. This could increase the number of effective approaches, implement them in culturally related health strategies, and target them to the personal values, needs, and resources of the highly heterogeneous populations of patients. As an example, India is mitigating the disease burden of NCDs by launching a National Health program that includes AYUSH systems [7].

The basis for such integrated endeavors is transparency of efficacy, effectiveness, safety, ethics, economics, and understandability of the healthcare strategies, which are the goals of evidence-based healthcare (EBHC): decision-making should be based on evidence, clinical expertise, and patients' values [8–10]. In pursuit of this goal, the development of new health technologies is driven by systematic research. Medicine, however, consists of many interventions, procedures, and treatment systems that have existed since long before the principles of EBHC were introduced. This applies not only to surgical, pharmacological, and nonpharmacological interventions and general care principles, but also to T&CM (traditional Chinese medicine and Ayurveda, for instance, have existed for thousands of years); self-help approaches (like teas, baths, and wraps); healthy and disease-preventive lifestyles; and integrative medicine (IM) overall.

IM is an umbrella concept, still being developed for the modern and evidence-informed integration of traditional, natural, mind-body, and complementary treatments with conventional medicine. IM systems share the following characteristics: emphasis on salutogenesis, the “natural healing power” of the organism; a holistic understanding of the human being, incorporating physical, mental, emotional, spiritual, and social issues; a focus on lifestyle modifications; extensive use of nonpharmacological interventions; strong emphasis on the therapeutic relationship between practitioner and patient; shared clinical decision-making supported by evidence; and the use of both conventional and complementary treatments [11, 12]. T&CM or IM systems comprise whole healthcare systems that have a distinct, unique perspective on nature, the human organism, and disease and derive their therapies accordingly [13]. Some T&CM interventions have received wide attention in medicine, e.g., *Artemisia annua* (Nobel Prize 2015 [14]), mind-body medicine techniques like meditation [15], yoga [16, 17], acupuncture [18], and many modern medicines derived from natural products that were first used in a traditional medicine context [1–3].

T&CM methods are broadly investigated, further developed, tested, and verified using scientific methods [2, 3, 18–20] that are supported by research networks (e.g., WHO, CAMbrella, NCCIH, SIO, ISCMR, ACIMH) [1, 21–28]. The Cochrane library lists more than 460 Cochrane reviews and more than 26,000 randomized controlled trials (RCTs) on T&CM [29]. These often mimic conventional mono-drug research investigating specific efficacy with explanatory placebo-controlled RCTs. However, given the specific challenges of investigating complex therapies as well as patient-centered care, there is currently a shift toward pragmatic research targeting comparative effectiveness of interventions as they are practiced in real-life situations. Furthermore, the importance of patients' subjective experiences is increasingly recognized as accountable and as necessary measures in health intervention evaluations. Also patient's healthy resources and values are seen as essential to promote a healthy lifestyle, reduce risk factors, and support compliance. These considerations lead to an increased use of qualitative research to explore patients' views and needs. They also lead to the construction of questionnaires that assess dimensions of health care that are of real concern for patients. A mixed-methods approach is pursued to gather information from multiple sources [1, 9, 21–28, 30–35].

Anthroposophic Medicine (AM) is one of the whole healthcare systems of IM [36]. It is based on a holistic, system-oriented understanding of man and nature, including disease and treatment. Its organismic concept consists of four levels (physical organization, life processes, soul, and spirit) and three constitutional systems (nerve-sense, metabolic-limb, rhythmic). AM is embedded in countrywide care systems, secondary and tertiary care hospitals, primary health centers, and private medical practices [36]. It applies medicines derived from plants, minerals, and animals; nursing procedures like rhythmical embrocations, baths, and wraps; arts therapies like music, painting, and sculpture; movement (eurythmy) therapy; physiotherapies such as rhythmical massage; lifestyle recommendations associated to AM philosophy concerning nutrition, agriculture, education; and meditation and mindfulness, psycho-spiritual counseling, and psychosocial support. AM care is provided by certified medical doctors, nurses, therapists, midwives, psychotherapists, and nutritionists [36–38]. AM education is provided by specific schools, universities, and other academic institutions [36].

AM has been widely investigated [36, 39, 40]; however, additional research activities are needed: owing to the goals of EBHC; for research-driven innovation and development in AM therapies and strategies; and to assess whether certain AM approaches can contribute to the management of significant healthcare problems, particularly chronic NCDs. These approaches include specific treatments such as certain nursing applications (to treat, for instance, insomnia, anxiety, chronic pain, osteoarthritis) [41, 42]; herbal extracts (e.g., for skin diseases, cancer, maternity care, and atopic diseases) [43, 44]; and eurythmy therapy (e.g., for chronic pain, mental conditions, high risk of falling among the elderly) [45]. They also include multimodal concepts (e.g., for fatigue and other quality-of-life issues in chronic NCDs and chronic infections) [46, 47]; models of patient-centered care (e.g., for pediatric diabetic care or depressive disorder) [48–50]; community care (e.g., for chronic pain with associated multimorbidity) [51]; strategies for dealing with fever (http://warmuptofever.org/en/) [52]; prevention strategies (for atopic diseases and allergies) [53, 54]; and support of self-efficacy [55] and perspectives on both the patient's and the care provider's needs [56, 57].

The investigation of a whole healthcare system like AM [13] entails a number of specific challenges:

- The patient-centered vs disease-centered approach, which is a hallmark of AM with its strong focus on individual resources of the patients and their psychological, biographical, and spiritual needs, and on shared decision-making and support of self-efficacy. This approach shifts the focus from study methods that assess effectiveness of therapies for average patients sharing a particular diagnosis; rather, it addresses the question of whether the therapy is effective for this individual patient with (potentially) several diagnoses (in theory, highly individualized patient-centered care can be tested as a black box in pragmatic comparative trials, but these trials, in addition to their exceeding complexity, lose explanatory power, transparency, and transferability of the results) [58].
- The multimodal approach, applying several interventions (including conventional and other T&CM methods) in the same patient, depending on the condition. This addresses the complexity of chronic NCDs in particular and the frequent concomitant diseases.
- The large diversity of treatments: about 1000 medicinal products and medicinally used natural substances [59], as well as therapeutic approaches like nursing approaches, arts or movement therapies, and counseling. This by far exceeds the single-component efficacy testing with one or two clinical trials each.
- The limited number of patients “fitting into” the trials while accepting a standardized care allocated randomly and who are not already recruited by competing trials [60–62]. Also, the limited acceptance of randomization by AM care providers [62] restricts the conductibility of large trials.
- The high costs of trials (confirmatory drug trials costing 11–53 million US$, on average [63]) and sparse funding possibilities (commercial interest restricted to a few remedies, rare public funding, most research being supported by foundations, philanthropic engagement or personal commitment of researchers). This necessitates an efficient use of resources.
- AM care is often perceived as a therapeutic process, a “healing journey” shared by patient and practitioners stemming from a spiritual “commitment” [64] and not to be disturbed by a clinical trial design. Specifically, as these “joined healing journeys” may reveal perspectives for addressing unmet needs of severely ill patients [65, 66], evaluation designs should preserve or even uncover preferences, individualization and intercultural experience, and potential effects related to practitioner-patient communication and rapport.

Given this complex situation and the challenges it presents, a strategic framework is sensible to test efficacy, effectiveness, and the cost-benefit ratio; to ensure safety and ethical principles as well as real-life application; to provide transparency and explore patients' needs, views, experiences, and the public's interest; and to contribute potential solutions for health challenges such as chronic NCDs. To develop this research strategy, a consensus process was chosen in order to incorporate different views, expertise, and resources.

## 2. Development of a Consensus-Based Research Strategy: Methodology

In developing the research strategy, we pursued a three-phase consensus process adapted to the *Guidance for Developers of Health Research Reporting Guidelines* [67, 68]: This consisted of (1) premeeting literature reviews, firstly on recommendations for investigating whole healthcare systems, and secondly on which objectives, to what extent, have been investigated on AM up to now; this was followed by interviews with key stakeholders to supplement or revise items of the research strategy, to include rationale and supporting references, and to tailor the strategy to AM research; (2) face-to-face consensus meetings for further developing and tailoring the strategy; and (3) postmeeting feedback followed by finalization.

### 2.1. Phase I

Key items of the strategy were developed from first literature review. They were presented to, discussed with, and supplemented by 162 key stakeholders in the field who fulfilled at least one of the following criteria: members of AM research council; representatives of AM at academic institutions, AM physicians, or members of patients associations; researchers in the field with different expertise; AM nursing and nursing scientists; arts therapists; representatives of AM supporting foundations; pharmacists (including AM-related pharma executives and research or medical directors); AM contact persons for regulatory agencies; and medical educators (including lecturers with university academic appointments). Stakeholders included healthcare practitioners employed in a conventional non-AM medical setting, and medical directors of units/departments within a conventional setting where AM is being newly implemented. Countries represented were Germany, Italy, Switzerland, Great Britain, Hungary, Sweden, Slovenia, Spain, South Korea, Netherlands, Israel, Russia, USA, Brazil, Chile, Peru, Argentina, and India.

### 2.2. Phase 2

The face-to-face consensus meeting at the International Research Council in Dornach, Switzerland (September 2017), included 25 participants. The meeting began with a review and discussion of the recommendations elicited during the literature review and interviews of Phase 1. These recommendations were clarified, further justified, modified, or new details were added. Additionally, the strategy was presented and discussed at two conferences in Filderstadt, Germany (May 2017), and Dornach, Switzerland (March 2018), including 45 and 107 international experts on AM research and practice, respectively.

### 2.3. Phase 3

The draft strategy was refined and sent for review to the AM Research Strategy Group (*n* = 48). The finalized strategy incorporated the feedback from this group.

## 3. Results: An Integrative Research Strategy

Just as AM covers many fields in healthcare, nearly all medical specialties, in different settings (outpatient, inpatient; primary, secondary, tertiary care; private practice or within the public health insurance system; acute and chronic conditions; medical prevention; health education and pedagogy) with the help of a wide variety of healthcare providers and training specialties, the field of active research is widespread.

Therefore, an integrative evaluation strategy is seen as sensible and has already been implicitly pursued. A reasonable amount of methodologically rigorous confirmatory RCTs on exemplary therapies and indications should be conducted. However, the majority of interventions should be assessed in system evaluations and smaller studies. Research on biomedical, physiological, pharmacological, psychological, anthropological, and nosological issues provides insight into treatment and care processes and also into patients' perspectives, goals, and achievements. The different sources of information around this strategy address individual interventions and also the complexity, different core aspects, and challenges of the whole healthcare system and the patient-centered approach. Loss of important information using only one design is minimized, as the specific focus and strength of each design complement those of others. Thus, by merging different designs and results, a comprehensive “evidence house” will be possible, with different parts serving as pieces of a puzzle to complete a whole picture.

The strategy could be a framework for

- Researchers investigating AM and T&CM, assessing or discussing AM, collaborating internationally
- Care providers participating in, supporting, informing, or presenting research
- The medical community in general, professional organizations, medical directors for information, transparency, dialogue, and decision-making
- Authorities, regulating AM
- Health policy and prevention policies implementing research results
- Private and public funders, pharmaceutical companies funding research
- Patients and their relatives and representatives, advocacy groups
- Students and educational organizations (e.g., universities) with interest in T&CM
- The public, civil organizations, via public relations and journalism

The methodological aspects of the strategy are outlined in the following section, supplemented by examples of previous and current AM research. The details are exemplary and will have to be adapted based on resources, research results, research methods, quality standards and administrative requirements, healthcare issues and health policy, funding policies, and the resources and interests of researchers.

The strategy refers to the research on (I) efficacy/effectiveness; (II) safety; (III) economics; (IV) evidence synthesis; (V) methodological issues; (VI) biomedical, physiological, pharmacological, pharmaceutical, psychological, anthropological, and nosological issues as well as innovation and development; (VII) patient perspective and involvement, public needs, and ethics; (VIII) educational matters and professionalism; and (IX) disease prevention, health promotion, and public health.

### 3.1. Efficacy/Effectiveness

#### 3.1.1. Evaluation of the Multidisciplinary Health System as a Whole, or of Complex Multimodal Therapy Concepts

A system evaluation, rather than assessing a specific remedy or treatment modality, approaches the entire complex, multidisciplinary, and multimodal treatment procedure. It is a “black box” approach to the whole treatment system or to specific multimodal therapy concepts and does not primarily differentiate the effects of the singular interventional elements. System evaluation studies assess several interventions that are simultaneously applied (for instance [46]) and/or individually tailored (for instance [62, 69, 70]). These studies can investigate established multimodal AM treatment concepts for relevant conditions (like rheumatoid arthritis [71] or cancer-related fatigue [46]) applied across all patients, and individually adapted to meet the patients' strengths and limitations. Outcomes can be disease-specific or more general PROMs (e.g., health-related quality of life) or patient-generated (e.g., MYMOP [72]). Such studies can also investigate the whole system of AM, for instance in patients with one or more chronic conditions (e.g., [69, 70, 73]), but also with acute diseases (e.g., [62]), diagnosed, treated, and counseled within the AM understanding of man and nature. Hereby, the healthcare providers' diagnostic and therapeutic abilities are also a major part of the “black box.” Depending on the conditions included, possible outcomes can be more generic, like a disease score or survival (e.g., in cancer), or patient-generated, or health-related quality of life, but also disease-specific outcomes in subgroups with a specific condition (as in the Anthroposophic Medicine Outcome Study, AMOS [69, 70]). These studies can also investigate certain care models, particularly for chronic diseases (e.g., depression [50] or community care models [51]), or specific patient-centered care models (like patient-centered diabetic care [48, 49]). The evaluation methods acknowledge the multilevel nature of many diseases and the individual presentation of diseases in most patients and the necessity for a multimodal and individualized therapeutic approach [1, 9, 21–28, 30–34, 74]. For such evaluations, different study designs can be chosen depending on the patient groups, interventions, controls, and contexts: comparative effectiveness studies, observational research (potentially using a bias suppression analysis or systematic outcome comparison as used in AMOS [75, 76]), experimental designs, pragmatic trials [69, 70], preference-based trials, quasiexperimental designs (using for instance interrupted time-series, instrumental variable analysis, regression discontinuity analyses, as reviewed in [77, 78]), or health services research [79]. In several of these designs, also matched-pair comparisons, synthetic controls and other regression techniques can be applied.

These evaluations require a good deal of methodological competence, knowledge, experience, and collaboration. Detailed a priori planning in this field is limited as it depends on the individual researcher and on opportunities to conduct such studies and available funding. A prespecified plan for the analysis and public protocol registration is strongly encouraged, whenever possible, using common databases such as ClinicalTrials.gov.

#### 3.1.2. Exemplary Interventions in Certain Indications: Confirmative Randomized Clinical Trials

A variety of specific AM interventions are well established and widely regarded as beneficial in certain health conditions. They may also support, independently from the AM treatment context, the effective treatment of major healthcare conditions (e.g., allergies, skin diseases, reducing nonindicated antibiotic prescriptions and resistance, chronic pain, mental diseases, risk of falling in elderly, and other chronic NCDs). The efficacy, safety, and efficiency of exemplary interventions should be investigated in a reasonable and feasible number of sufficiently powered confirmatory RCTs, with the potential goal of the interventions being implemented in treatment guidelines.

To be worthwhile for an investigation in confirmative RCTs, interventions need to meet certain conditions: they have to have a rationale for the improvement of a relevant health condition. This should be supported by good empirical data from clinical, physiological, or exploratory studies, in order to design the trial, define outcomes, follow-up periods, control intervention, and calculate the sample size. This also refers to issues of application form, dosage, and duration. They should be applied in a health condition with a need for further interventions, e.g., when standard interventions have limited efficacy, are not well accepted by patients, or are better accepted with a cointervention (e.g., mistletoe extracts in cancer or cancer-related symptoms [80, 81], NCT02948309, NCT02106572); dermatology [44]; eurythmy therapy in cases of high risk of falling (DRKS00016609); and nursing procedures in pain, obstipation, nausea, sleep disturbance, anxiety, and others [82, 83]). Confirmative RCTs are often large, conducted in multicenter settings, as a collaborative effort of experts in different fields (including patient representatives) and guided by various guidelines, standards, and national requirements. These trials are very expensive and require appropriate funding and clinical infrastructure, both of which are in relatively short supply in AM.

#### 3.1.3. The Wide Range of Remaining Interventions and Patient-Centered Care Strategies: Well-Conducted Small Trials and Observational Studies, High-Quality Case Reports and Series, Subgroup Analyses from Whole-System Studies, and Health Service Research

The large quantity of AM interventions and their use within a patient-centered approach (about 1000 medicines, but also nursing procedures, arts therapies, eurythmy therapy, massages, embrocation, packs, teas, dietary advice, lifestyle counseling, therapeutic talks regarding emotional, biographical, social, and spiritual issues, and educational methods) exceed the current capacity to assess these interventions in large confirmative trials. Therefore, the majority of interventions should be (and have been) investigated or transparently presented, partly in smaller formats but still with high methodological quality: for instance, observational studies and small clinical trials; case series and case reports; subanalyses from large studies [70, 84]; matched-pair comparisons (AM vs not AM-treated patients); health service research (e.g., clinical registries); or research syntheses from other similar interventions (e.g., non-AM Arnica preparation). Many methodological elements described above for the evaluation of health systems can also be applied.

Altogether, many of these methods, supported by quality standards and guidelines, are less demanding, complex, and costly and can be conducted as smaller research projects. Every effort has to be done to enhance quality and transparency and reduce the risk of bias. Still, these designs will often not provide causal evidence and are frequently subject to bias. These studies will often make use of “real-life data,” with the primary focus sometimes switching to the point of care, providing insights into therapeutic procedures like patient-centered care [48] or addressing specific, unique conditions. [42, 85] Some of these methods will be influenced by future specific or general developments [86–88].

### 3.2. Safety

With regard to safety, a variety of objectives and methods can provide information and have been used for investigations in AM: a key source for safety data are adverse events and tolerability assessed within clinical trials and studies. Another, much wider source for safety information is pharmacovigilance studies and health service research [59, 89]. Many side effects of interventions, also rare ones, are captured with case reports and reports from authorities [39]. Vulnerable populations, like pregnant women, children, or elderly, need particular attention and research—in clinical trials, observational or pharmacovigilance studies, or specific registries. Specific safety issues (e.g., pharmacological interaction of AM-drugs with conventional drugs or with other AM or T&CM drugs [90–92]) may need a focused investigation using different methodologies.

Besides these classic assessments of untoward effects of interventions, also other safety issues are related to the whole field of T&CM as well as AM and can be investigated with different methodologies: for instance the impact of AM on patients' adherence to conventional medicine treatments (e.g., compliance with scheduled chemotherapy protocol [93]) and associated treatment effectiveness or the impact of AM on decreasing unjustified overuse of conventional drugs (nonindicated antibiotics prescriptions, analgesics overuse, etc. [51, 62, 94, 95]), to reduce drug-associated adverse effects, morbidity, and health costs [59, 71, 96–98]. A further safety issue necessitating also exploratory research relates to patients who adopt “alternative” health belief models that they (wrongly) associate with AM, expressed by negation of evidence-based conventional treatments or prevention recommendations (e.g., certain vaccinations, antibiotics, chemotherapy, steroids).

For the various methods, standards and guidelines exist or have to be adapted. A large body of evidence is available [36, 39, 59] and cooperation with national drug agencies established.

### 3.3. Economics

The economic implications of therapeutic strategies should be evaluated [96–98]. Direct, indirect, and intangible costs have to be considered, as well as different perspectives (e.g., societal, patient, and health insurance), depending on the setting. Different evaluation techniques are available (e.g., cost-effectiveness analysis, cost-utility analysis, and cost-benefit analysis), cost-effectiveness analysis being the most common. RCTs should consider collecting cost data (e.g., inpatient care, outpatient care, etc.) and standardized quality-of-life metrics to allow an estimate of quality-adjusted life-years (QALYs). Real-life data (registries) can be used as well in context with good quality records. The extent of the evaluations should be such to capture all relevant differences regarding outcomes between the intervention and comparators; therefore, lifetime horizons using decision-analytic models are preferred [99–101]. Adopting Good Research Practices from ISPOR is highly recommended (http://www.ispor.org). Reporting of economic evaluations of AM should follow the standard Consolidated Health Economic Evaluation Reporting Standards (CHEERS) [102].

### 3.4. Evidence Synthesis, Systematic Reviews, and HTA Reports

The results of efficacy and effectiveness research and of safety and economic evaluations have to be systematically collected, reviewed, and assessed in their methodological quality and analyzed across individual studies. This applies to both single interventions on certain conditions and complex, multimodal or individualized therapeutic systems. The goal would be that the strengths of and specific information gleaned from the different designs and studies complement each other so that the compiled data and results form a reasonable, informative, and transparent evidence house (which should then also include points 3.5.–3.7.). Modeling studies can be helpful in estimating the public health benefit and costs of AM based on data from primary or secondary sources to inform decisions on healthcare policy [103].

For clinical trials and observational studies, the methodology of systematic reviews and meta-analyses has been developed, and widely used methods as proposed by Cochrane and GRADE are available (http://www.cochrane.org). For other assessment designs, complex, integrative intervention models, case reports, etc., meta-methods like mixed-methods reviews, critical appraisal, and evidence-mapping remain to be fully considered or further developed. On a broader perspective, HTA reports can be useful [39, 40, 104].

### 3.5. Methodologic Issues

For many research designs, methods and standards have been developed, and guidelines are available to ensure quality and to define the applicability and generalizability of the results. For other designs, such as analyses and subanalyses, these methods have to be further developed. This concept relates specifically, for instance, to “whole system” and “complex intervention” research; bias reduction and systematic outcome comparison in large observational studies [75, 76]; case reports [105, 106] and their use (e.g., applicability, strengths, weaknesses, generalizability, causality, [107–109]) and their systematic assessment; health service research, care models, and issues related to clinical judgment and expertise [110], clinical decision analysis, individualizing and patterning of clinical responses [107, 108, 111], and “process-oriented research” [112].

The development of methods is carried out in close cooperation and consensus procedures with competent epidemiologists, statisticians, economists, methodologists, clinical researchers, and healthcare experts in related fields. An ongoing dialogue with decision makers, health professional organizations, and journal editors helps to incorporate their views and interests.

### 3.6. Biomedical, Physiological, Pharmacological, Pharmaceutical, Psychological, Anthropological, and Nosological Issues, as well as Innovation and Development

Research should unravel the working principles of the interventions, contribute to the transparency of the AM concepts of the human organism and of health and disease, relate these to other medical and scientific concepts, and further develop medical and healthcare strategies. This relates to AM remedies, to understand their molecular, epigenetic, cellular, biochemical, physiological, pathophysiological, immunological, neurological, psycho-neuro-immunological mechanisms involved, the social context, etc. [43, 113, 114]. The same accounts to nonpharmacological interventions, lifestyle changes, or counseling: do they have effects on physiological rhythms, epigenetics, the psychosocial level, or on individual mental-cognitive developmental processes? A key issue is specifics of dosage and application and other pharmacological questions as well as issues of pharmaceutical quality. As AM has a distinct and hierarchical concept of the human organism that extends into nosological and diagnostic categories, these diagnostic (practice) methods, for instance, the constitution types of AM, have to be further elaborated and validated. This also includes the development and validation of questionnaires in different fields [115, 116]. Altogether, the anthroposophic-anthropologic concept of the human being, nature, health, and disease (salutogenesis) and therapy is a large field for epistemological, conceptual, and experimental research. This includes the concepts of human beings as social individuals (bio-psycho-social-spiritual approach), of organisms as complex adaptive systems, and of emergent behavior [117–119]. Also, AM nursing models and concepts, widely established in practice, can be further investigated. Last not least, these fields connect to innovations and further development of interventions within AM health care [82, 83, 114].

### 3.7. Patient Perspective and Involvement, Public Need, and Ethics

The view from the goal—investigating patients' perspectives using AM healthcare systems or specific AM interventions, and involving patients in research—provides important information and is a key area in the research field today: patient and public needs, interests, and perspectives on AM are assessed by qualitative and questionnaire-based methods and systematic metaethnographic approaches [120–122]. For clinical research, patient-relevant and experience measurements are developed (PROMS and PREMS) [123]. First-person perspective studies, including biographic introspection, can give important insight in understanding the subjective dimension of disease. Patients are increasingly involved in the development of study designs and priority-setting (see http://www.invo.org.uk), also in AM (e.g., ENTAIER trial, DRKS00016609), which is another field of further development. Patient involvement and research also focuses on the development, evaluation, and implementation of patient empowerment, patient information, and decision-making material, as well as self-care programs using AM [49, 124, 125]. Another large field refers to the elements of ethics in AM healthcare professions and in applications of AM interventions in and outside AM and to contributions to the general ethics discussion. This includes general topics of medical ethics but also the important issue of informed consent, particularly with regard to missing evidence or to lacks in safety data, and the issue on how to deal with interventions or recommendations, which lack robust evaluation.

### 3.8. Educational Matters and Professionalism

Educational research provides important insights into the clinical trainings of healthcare professionals, the quality and issues of medical training, and the AM education, including medical students' possible contributions to patient-centered care [126, 127]. Furthermore, integration of AM (or parts of it) into established healthcare systems could be outlined and investigated, and the impact of AM courses on medical students' perspectives in pregraduate and postgraduate settings could be evaluated [128, 129]; furthermore, the impact of integrative medicine training on a mixed AM and non-AM group of practitioners, preferably in a multidisciplinary context, could be evaluated [130], as well as the influence of stress on burnout symptoms and empathy of care providers and their spiritual needs [57, 131]. Criteria for professionalism specific to AM or T&CM physicians [132] could be further adapted for all health professions.

### 3.9. Disease Prevention, Health Promotion, and Public Health

AM healthcare aims to understand and support the whole human being. Therefore, in addition to treating illnesses and symptoms, healthy development is supported during the entire lifetime (i.e., before, during, and after birth; during childhood, adolescence, adulthood, and end-of-life) and also during the development of emotional, cognitive, and spiritual competencies. A positive health concept is the goal [133]. Interdisciplinary work is pivotal and also includes consideration of pedagogy and agriculture and environmental aspects. Research in these areas will depend on research collaboration and networking on a large scale, including collaboration with, for instance, epidemiologists, healthcare insurers, and public health institutions. [53, 54, 134].

## 4. Discussion

This research strategy covers the large spectrum of a whole healthcare system. It encompasses an array of experimental and explanatory to observational and pragmatic designs, from preventive to palliative care, from intervention to the patient's perspective, from inpatient to outpatient care. The fields of basic and conceptual research and innovation also are a part to this strategy but are only roughly outlined. The strategy is based on the following: existing methodological discussion of investigating whole medical and healthcare systems including complex interventions [1, 9, 21–28, 30–34]; what is regarded as important by key stakeholders; and what research is actually currently pursued or planned. Future developments may further evolve and refine this strategy.

This strategy can provide a broad view of the different aspects of the whole medical and healthcare system of AM and can also support the development of specific interventions or healthcare concepts that may be relevant for healthcare in general. It also provides insight about patients' perspectives and needs and with regard to issues of education and professionalism. It offers a framework for different stakeholders in medicine, science, and the general public, and it may improve intercultural transparency. The pluralistic and integrative nature of the presented strategy portends that the whole body of results will present a more adequate perspective of the complex field of AM than the isolated parts would have.

The scope of the strategy does not specify the fields actually being the focus of research. We presume that managing and treating chronic NCDs and focusing on disease prevention will be of primary interest. Still, the actual focus depends on the individual researchers and clinicians; their institutions' interests, capabilities, and infrastructures; the related collaboration and networks; the potential benefit expected with the specific intervention under investigation; and also on public interest and the priority-setting of funders.

Four leading obstacles impede the promotion of AM research with high-quality methodology.

### 4.1. External Factors

Successful high-quality research will be impeded by **budget** limitations. However, even large studies are increasingly funded, and therefore an increasing rate of high-quality and clinically significant research will foster trust in AM research. **Inclination** of the scientific community to embrace explanatory rather than pragmatic trials and RCTs rather than observational and real-life studies will impede the successful conduction, funding, and publication of whole healthcare systems studies. However, there is an increasing interest in a broader spectrum of designs due to the complexity and individualization of medicine [9, 30–34, 86–88], and therefore, high quality of these studies as well as research on methodologic issue will be essential.

### 4.2. Internal Factors

These relate to a limited number of trained AM researchers and healthcare settings as well as to potential **reluctance** of some AM clinicians and healthcare providers to conduct, support, and participate in clinical research due to workload, skepticism about research, standardized care, and randomized treatment allocation that conflicts with the individualized, patient-centered approach they would like to employ. Close collaboration, consideration of clinicians' and healthcare providers' needs and constraints, and communication about the benefits and risks of research (e.g., presenting research projects and results at AM practitioners' conferences and integrating AM clinicians, healthcare providers, and patients in research planning and study design) may help to overcome these limitations. Still, a variety of research studies, specifically experimental, highly standardized designs like RCTs on a specific treatment in a certain disease, will have to be conducted outside the AM setting (e.g., NCT02948309 and NCT02106572).

### 4.3. Methodologic Factors

While for RCTs a variety of guidelines, standards, and requirements are well defined, other study types may have less rigor and less demanding quality criteria and guidelines. Therefore, quality and scrutiny parameters in planning, design, data quality, analysis, presentation, careful inferences, and general standards of good clinical research have to be strictly followed [135–137]. Commitment by researchers and review by ethical committees, funders, and journals still might not ensure high quality. Therefore, the development of further specific methodological-quality guidelines for researchers and funders and later assessments, as well as specific training, additional internal and external peer review, discussion within the broader network already in the planning phase, and continuous methodological dialogue and awards for high-quality projects, may support and improve this approach long-term. Furthermore, the assessments of such an area of integrative research studies, for instance with HTA reports, may become elaborate. This may necessitate further methodological developments to improve pragmatic and efficient meta-assessments.

The strength of this consensus-based strategy is the consideration of different dimensions of a healthcare system, the inclusion of a large variety of stakeholders like care providers, MDs from in and outpatient care with different specialties, directors of institutions or hospitals, pharmacists, patient representatives, funding bodies, and researchers with diverse expertise and intercultural aspects. Thus, the input is based on specific scientific knowledge as well as long practical experience with patient care, research projects, and extensive collaboration.

Still, this consensus strategy also has some limitations. It is a current view and does not foresee future developments, researchers, and stakeholders, which may modify some items of the strategy. It also does not foresee the availability of resources and funding. Therefore, the strategy is a matter of estimation and intention. Although the consensus process includes many, it does not include all relevant stakeholders outside AM, like public funders, journal editors, researchers conducting future systematic reviews and HTA reports on AM or investigating other whole healthcare systems, and authorities. However, the included stakeholders have multiple collaborations with these “external” stakeholders.

In conclusion, this strategy provides a wide spectrum of research that will assess many facets of a whole healthcare system pursuing patient-centered care. This may contribute to solutions for global health challenges, particularly with regard to chronic NCDs and health promotion. The culture of collaboration with other IM and non-IM methodologists and researchers is of great importance and value. Researchers investigating other integrative modalities such as traditional Chinese and Ayurvedic medicine as well as researchers investigating patient-centered care and patient-tailored treatment (e,g. Family medicine, palliative medicine, narrative-based medicine, and psycho-social-ethno literature, spiritual care research) are confronted with some or all of the challenges described in this article. Interdisciplinary and international collaboration effect more expertise and infrastructure for high-quality research projects. Furthermore, collaboration with other stakeholders in the healthcare system, at academic institutions, at professional and patient organizations, with associations and committees involved in guideline development and healthcare planning, research, and funding, will assist with purposeful, efficient, and high-quality research development.

## 5. Conclusion

T&CM, used worldwide and integrated into EBHC, can play an important part in health services, supporting health, and addressing chronic NCDs. Its focus is on patient-centered care, and it is linked to the cultural background and needs and values of patients. Transparency and information are provided by a strong and differentiated evidence base regarding benefit and implementation of T&CM approaches, assessing efficacy, effectiveness, safety, costs, modes of action, patient and public perspective, ethical issues, educational matters, professionalism, and healthcare procedures and concepts. A broad research strategy, as outlined for Anthroposophic Medicine, supports research and healthcare, transcultural understanding, and collaboration among different stakeholders of healthcare.

## Data Availability

The data are available from the public databases (e.g., PubMed/MEDLINE) or are available from corresponding author on reasonable request.

## Abbreviations

ABHC: Evidence-based healthcare
ACIMH: Academic Consortium for Integrative Medicine and Health
AM: Anthroposophic Medicine
AMOS: Anthroposophic Medicine Outcome Study
CAMbrella: European research network for complementary and alternative medicine
CHEERS: Consolidated Health Economic Evaluation Reporting Standards
ENTAiER trial: Randomized controlled trial on efficacy and safety of Eurythmy Therapy and Tai Chi in chronically ill elderly patients with high risk of falling
HTA: Health Technology Assessment
IM: Integrative Medicine
ISPOR: International Society for Pharmacoeconomics and Outcomes Research
ISCMR: International Society for Complementary Medicine Research
MYMOP: Measure Yourself Medical Outcome Profile
NCCIH: National Center for Complementary and Integrative Health
NCDs: Noncommunicable diseases
NCT: ClinicalTrials.gov registry number
PREMS: Patient reported experience measures
PROMS: Patient reported outcome measures
RCTs: Randomized controlled trials
SIO: Society for Integrative Oncology
T&CM: Traditional and complementary medicine
WHO: World Health Organization.
